# Associations of whole blood polyunsaturated fatty acids and insulin resistance among European children and adolescents

**DOI:** 10.1007/s00431-020-03636-1

**Published:** 2020-04-08

**Authors:** Sarah Marth, Claudia Börnhorst, Kirsten Mehlig, Paola Russo, Luis A. Moreno, Stefaan De Henauw, Toomas Veidebaum, Dénes Molnár, Michael Tornaritis, Patrizia Risé, Maike Wolters, on behalf of the IDEFICS and I.Family consortia

**Affiliations:** 1 Leibniz Institute for Prevention Research and Epidemiology – BIPS, Achterstr. 30, 28359 Bremen, Germany; 2 Sahlgrenska School of Public Health and Community Medicine, University of Gothenburg, Gothenburg, Sweden; 3 Institute of Food Sciences, National Research Council, Avellino, Italy; 4 GENUD (Growth, Exercise, Nutrition and Development) Research Group, Instituto Agroalimentario de Aragón (IA2), Instituto de Investigación Sanitaria Aragón (IIS Aragón), Centro de Investigación Biomédica en Red Fisiopatología de la Obesidad y Nutrición (CIBERObn), University of Zaragoza, Zaragoza, Spain; 5 Department of Public Health, Faculty of Medicine and Health Sciences, Ghent University, Ghent, Belgium; 6 National Institute for Health Development, Tallinn, Estonia; 7 Department of Pediatrics, Medical School, University of Pécs, Pécs, Hungary; 8 Research and Education Institute of Child Health, Strovolos, Cyprus; 9 DISFARM, Department of Pharmaceutical Sciences, University of Milan, Milan, Italy

**Keywords:** Children, HOMA, Insulin resistance, n-3 fatty acids, n-6 fatty acids, Polyunsaturated fatty acids

## Abstract

This study aims to examine the association of whole blood n-3 and n-6 polyunsaturated fatty acids (PUFA) with insulin resistance (IR) in children. Whole blood fatty acids were measured in 705 children aged 2–9 years of the European IDEFICS/I.Family cohort using gas chromatography in units of weight percentage of all detected fatty acids (%wt/wt). IR was determined by the Homeostasis Model Assessment for IR (HOMA). Mixed effect models were used to assess the associations between selected baseline PUFA and HOMA *z*-scores at baseline and after 2- and 6-year follow-ups using models with basic and additional confounder adjustment as well as stratified by sex and weight status. In the basic model, α-linolenic (*β* = 1.46 SD/%wt/wt, *p* = 0.006) and eicosapentaenoic acid (*β* = 1.17 SD/%wt/wt, *p* = 0.001) were positively associated with baseline HOMA *z*-score. In the stratified analyses, α-linolenic acid was positively associated with HOMA *z*-score in girls only (*β* = 1.98 SD/%wt/wt, *p* = 0.006) and arachidonic acid was inversely associated with baseline HOMA in thin/normal-weight children (*β* = − 0.13 SD/%wt/wt, *p* = 0.0063). In the fully adjusted model, no statistically significant associations were seen.

*Conclusions*: Our overall results do not indicate a protective role of higher blood n-3 PUFA or an adverse role of higher blood arachidonic acid proportion on the risk of IR.

**Table:** 

**What is Known:** *•Intervention studies reported a beneficial effect of n-3 PUFA supplementation on insulin resistance compared with placebo while observational studies in cildren are inconclusive.* *•Studies have shown a positive association of n-6 arachidonic acid and insulin resistance indicating an adverse role of arachidonic acid.*	
**What is New:** *•Cross-sectional and longitudinal analyses based on circulating blood fatty acid concentrations in a large cohort of European children and adolescents.* *•Overall results do not support a protective role of n-3 PUFA or an adverse role of arachidonic acid in insulin resistance.*

**Electronic supplementary material:**

The online version of this article (10.1007/s00431-020-03636-1) contains supplementary material, which is available to authorized users.

## Introduction

The worldwide obesity epidemic has resulted in a rise of insulin resistance (IR) that is already observed in childhood and adolescence. IR is often accompanied by further components of the metabolic syndrome and tends to track into adulthood [1]. Polyunsaturated fatty acids (PUFA), particularly n-3 PUFA, may beneficially influence insulin sensitivity (IS) whereas the n-6 PUFA, arachidonic acid (AA), may have an adverse effect [2, 3]. Accordingly, in adults with overweight or obesity, a higher dietary n-6:n-3 ratio has been associated with increased insulin resistance, measured as Homeostasis Model Assessment for insulin resistance (HOMA) [4]. Correspondingly, in adolescents with overweight and obesity who participated in a weight loss program, the reduction in n-6 PUFA was observed to be directly related to reduced glucose concentrations [5]. Further, AA in adipose tissue triacylglycerol was found to be positively associated with HOMA in healthy, non-obese children [2]. Interventional studies indicated that supplemental dietary n-3 PUFA improve insulin sensitivity [6, 7]. Moreover, n-3 PUFA status of children as measured in serum, [8] serum phospholipids [9], whole blood [10], and erythrocytes [11] has been shown to be inversely associated with IR, although two studies investigating n-3 PUFA in plasma phospholipids and plasma lipids did not confirm this relation [12, 13]. In particular, in studies with mostly small sample size, the n-3 PUFA α-linoleic acid (ALA) [14], eicosapentaenoic acid (EPA) [8], docosahexaenoic acid (DHA) [10], and the sum of EPA+DHA [11], as well as AA [2], have been linked to IR and HOMA. Large longitudinal studies in children with data on fatty acids and IR from biosamples are scarce. Therefore, our longitudinal study investigated the cross-sectional and longitudinal associations between the above-mentioned PUFA and HOMA for the first time in a large cohort of young children across Europe.

## Materials and methods

**Fig. 1:**
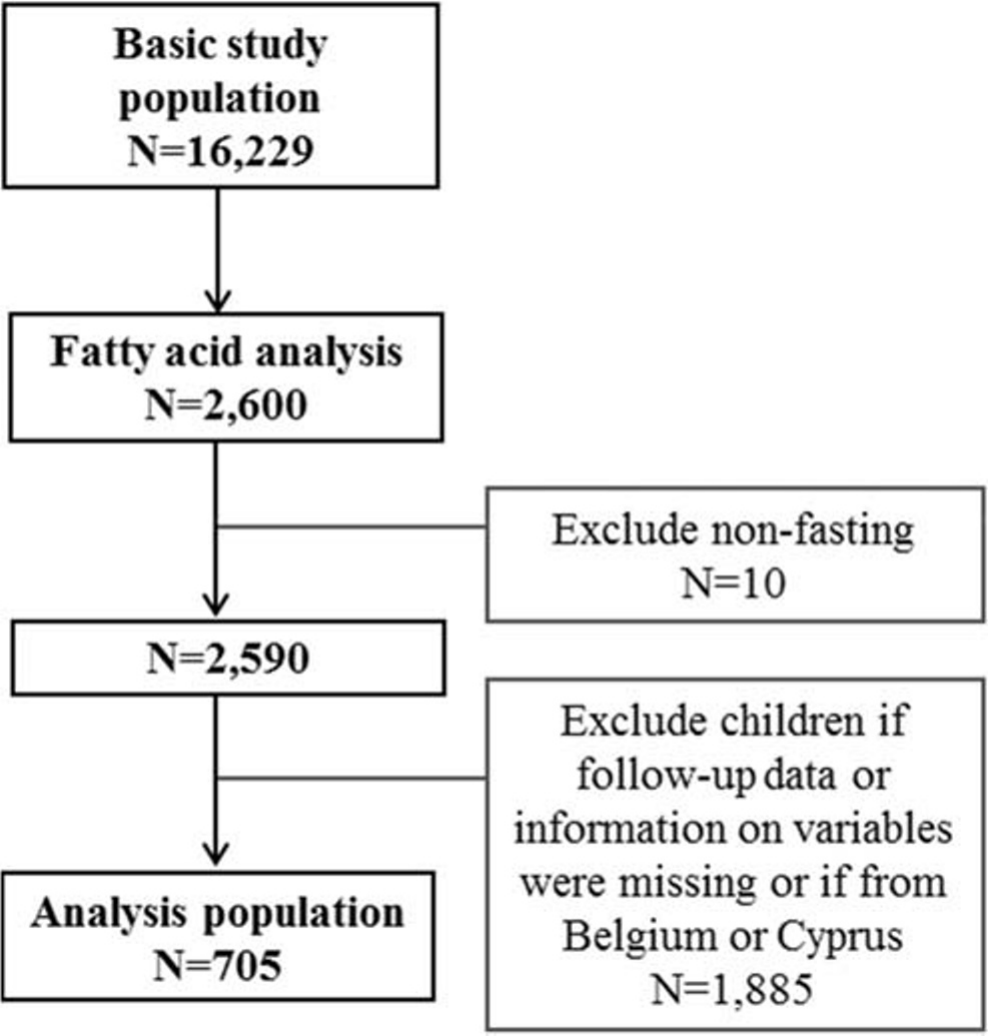
Flow chart of the inclusion and exclusion of IDEFICS/I.Family participants

A subsample of the IDEFICS/I.Family cohort was included. The baseline survey (T0) took place in 2007/2008 with follow-up examinations after 2 (T1) and 6 (T3) years. Ethical approval was obtained from all participating study centers. The fatty acid blood profiles of 2600 children were analyzed and included as weight percentage of all fatty acids (FA) detected (%wt/wt). All children whose FA data were available at baseline and who participated in at least one follow-up examination were included in the present analysis (*N* = 705; 705 T0, 571 T1, and 342 T3; Fig. 1). HOMA was calculated as fasting insulin (μǀU/ml) × fasting glucose (mmol/l)/22.5 [15]. For the description of the characteristics of the study population, children with a HOMA greater or equal to the 90th percentile (≥P90) were considered to be insulin resistant or at risk. Mixed effect models were used to assess the associations between baseline PUFA (ALA, EPA, DHA, EPA+DHA, AA) and continuous HOMA *z*-score measured at baseline and 2- and 6-year follow-ups. All models were run with a basic adjustment (age, sex, country of residence, and intervention vs. control region) as well as adding further covariates (fully adjusted model; see Table 1). A *p* value of 0.01 was considered to be statistically significant. Detailed information on the assessment methods of fatty acids, HOMA, anthropometric measures, and covariates as well as statistical analysis methods can be found in the Supplementary Material.

**Table 1:** Associations of polyunsaturated fatty acids measured at baseline with repeated measurements of HOMA *z*-scores at baseline and after 2 years and after 6 years of follow-up estimated on basic and fully adjusted mixed effect models

Fatty acid	Time since baseline in years	Basic		Full adjustment^†^	
		*β*	*p* value	*β*	*p* value
20:4n-6, AA	0	− 0.044	0.2477	− 0.074	0.0544
	2	0.069	0.0706	0.025	0.5108
	6	0.030	0.6142	− 0.001	0.9879
18:3n-3, ALA	0	**1.457**	**0.0062**	0.718	0.1220
	2	0.197	0.7537	− 0.251	0.6437
	6	− 0.235	0.7572	− 0.481	0.4835
20:5n-3, EPA	0	**1.172**	**0.0012**	0.645	0.0608
	2	0.576	0.1786	0.286	0.4201
	6	− 0.816	0.2357	− 1.159	0.0728
22:6n-3, DHA	0	0.011	0.9213	0.066	0.5159
	2	0.133	0.2387	0.184	0.0654
	6	− 0.007	0.9687	− 0.022	0.8996
20:5n-3+22:6n-3 (sum of EPA+DHA)	0	0.071	0.4565	0.087	0.3182
	2	0.132	0.1800	0.159	0.0651
	6	− 0.055	0.7311	− 0.082	0.6004

^†^The basic model was adjusted for age, sex, country of residence, and control vs. intervention region. The fully adjusted model was further adjusted for birth weight, BMI *z*-score, pubertal status, family history of diabetes mellitus type 2, being a member of a sports club, consumption frequency of sugar/refined carbohydrates, time spent with audio-visual media, maximum ISCED level of parents, weight percentage of the sum of total SFA and total MUFA of total fatty acids

As highlighted in bold, a *p* value of 0.01 was used as the level of statistical significance

*AA*, arachidonic acid; *ALA*, α-linolenic acid; *DHA*, docosahexaenoic acid; *EPA*, eicosapentaenoic acid; *HOMA*, Homeostasis Model Assessment for insulin resistance; *ISCED*, International Standard Classification of Education; *MUFA*, monounsaturated fatty acids; *SFA*, saturated fatty acids

## Results

Supplementary Table 1 shows that HOMA of ≥P90 was more prevalent in girls and in children who had no sportsclub membership, who spent more time with audio-visual media (except for T3), who had a familial history of diabetes, and whose parents had a lower education level. Further, children with HOMA of ≥P90 were more likely to have been obese, compared with children without IR (47% vs. 12% at T0, 48% vs. 9% at T1, and 28% vs. 14% at T3). Regarding the association of different PUFA with HOMA *z*-scores at baseline and after 2 and 6 years of follow-up, ALA (*β* = 1.46, *p* = 0.0062, i.e., 1-unit (%wt/wt) increase of ALA was associated with a 1.46 SD higher HOMA *z*-score) and EPA (*β* = 1.17, *p* = 0.0012) were positively associated with HOMA at baseline in the basic model. These associations weakened over time, as reflected by lower and negative *β*-values for HOMA *z*-scores at T1 and T3 (Table 1). Similar associations were observed in the fully adjusted models. These were however not significant. In the basic model stratified by sex, ALA (*β* = 1.98, *p* = 0.0058) and EPA (*β* = 1.24, *p* = 0.0057) were positively associated with HOMA in girls but not in boys (Supplementary Table 2). When stratified by weight status in the basic model, AA was inversely associated with HOMA *z*-score (*β* = − 0.131, *p* = 0.0063) at baseline in thin/normal-weight children. A similar association was observed in the fully adjusted model (*β* = − 0.134, *p* = 0.0169). This was however only marginally significant (Supplementary Table 3).

## Discussion

Our study was based on an exceptionally large number of measurements and provides cross-sectional and longitudinal data on the association of whole blood PUFA with HOMA in a cohort of 705 European children. In general, our findings do not indicate a role of ALA, EPA, DHA, EPA+DHA, or AA in IR. Contrary to expectations, an adverse effect of higher ALA and EPA in the total analysis sample was observed in our basic model. However, as these associations disappeared in the fully adjusted models, they may be partially explained by confounding, e.g., by covariates such as BMI or the consumption frequency of sugar and refined carbohydrates. Although IR in adolescents is influenced by various factors, obesity has the strongest effect [16]. Therefore, we considered BMI a confounder and not a mediator. Our results are in line with those of a Danish cross-sectional study including 713 children aged 8–11 years, which did not find an association between whole blood EPA and HOMA [10]. Another study including 120 adolescents with normal weight and overweight also did not find an association between n-3 and n-6 PUFA levels of plasma phospholipids and cholesterol esters and HOMA [13]. Our results in the basic model indicating an adverse effect of EPA are in line with a study involving 56 obese Mexican children, which reported significantly higher serum levels of EPA in insulin-resistant compared with non-insulin-resistant children [8]. As in our population, no association of DHA in plasma phospholipids with HOMA was observed in yet another study on 32 children with obesity [12]. In contrast, in the Danish study [10] and in a study including 10 adolescents with obesity and 15 normal-weight adolescents reporting DHA in serum phospholipids [9], DHA was inversely associated with HOMA. Concordantly, in a small Australian study including 24 children with and 24 without obesity, the sum of EPA and DHA levels of erythrocytes was moderately inversely associated with HOMA [11].

While other studies indicate an adverse role of AA, we unexpectedly observed a beneficial role of higher AA in thin/normal-weight children in our basic model. In a Spanish study with 83 healthy non-obese children, AA in adipose tissue triacylglycerols was positively associated with HOMA [2]. The reduction of plasma n-6 PUFA after a weight loss program was also reported to be associated with improved fasting glucose in adolescents [5]. Accordingly, in adult males, EPA/AA ratios of erythrocytes and EPA were negatively associated with IR in subjects with metabolic syndrome [3]. Additionally, among adults with and without diabetes, plasma AA was found to be highest among those with diabetes compared with subjects with normal glucose tolerance [17]. Besides differences in the methods applied (see Supplementary Material), a possible explanation regarding why we did not observe the expected associations in our study compared with others may be the differences in desaturase activity between study populations. In previous IDEFICS/I.Family study analyses, we observed that higher estimated delta-6 desaturase activity was associated with an increase of IR [18] and that genetic variations in the *FADS1* gene, which affects delta-5 desaturase, influenced whole blood AA and EPA levels [19].

In conclusion, taking relevant confounders into account, our overall results do not point to an association between n-3 PUFA or AA and IR. Given the high prevalence of obesity in children with IR, prevention programs of IR need to focus on obesity as a known main risk factor.

## Electronic supplementary material

ESM 1 (DOCX 88 kb)

## Abbreviations

ALA: α-Linolenic acid
BMI: Body mass index
DHA: Docosahexaenoic acid
EPA: Eicosapentaenoic acid
FA: Fatty acids
HOMA: Homeostasis Model Assessment for insulin resistance
IDEFICS: Identification and prevention of dietary- and lifestyle-induced health effects in children and adolescents
IR: Insulin resistance
IS: Insulin sensitivity
ISCED: International Standard Classification of Education
PUFA: Polyunsaturated fatty acids
