# Correction to: Associations of whole blood polyunsaturated fatty acids and insulin resistance among European children and adolescents

**DOI:** 10.1007/s00431-021-04264-z

**Published:** 2021-09-24

**Authors:** Sarah Marth, Claudia Börnhorst, Kirsten Mehlig, Paola Russo, Luis A. Moreno, Stefaan De Henauw, Toomas Veidebaum, Dénes Molnár, Michael Tornaritis, Patrizia Risé, Maike Wolters, the IDEFICS, I.Family consortia

**Affiliations:** 1 Leibniz Institute for Prevention Research and Epidemiology – BIPS, Achterstr. 30, 28359 Bremen, Germany; 2 Sahlgrenska School of Public Health and Community Medicine, University of Gothenburg, Gothenburg, Sweden; 3 Institute of Food Sciences, National Research Council, Avellino, Italy; 4 GENUD (Growth, Exercise, Nutrition and Development) Research Group, Instituto de Investigación Sanitaria Aragón (IIS Aragón), Centro de Investigación Biomédica en Red Fisiopatología de La Obesidad Y Nutrición (CIBERObn), Instituto Agroalimentario de Aragón (IA2), University of Zaragoza, Zaragoza, Spain; 5 Department of Public Health, Faculty of Medicine and Health Sciences, Ghent University, Ghent, Belgium; 6 National Institute for Health Development, Tallinn, Estonia; 7 Department of Pediatrics, Medical School, University of Pécs, Pécs, Hungary; 8 Research and Education Institute of Child Health, Strovolos, Cyprus; 9 DISFARM, Department of Pharmaceutical Sciences, University of Milan, Milan, Italy

## Correction to: European Journal of Pediatrics (2020) 179:1647–1651 https://doi.org/10.1007/s00431-020–03636-1

The article “Associations of whole blood polyunsaturated fatty acids and insulin resistance among European children and adolescents”, written by Sarah Marth, Claudia Börnhorst, Kirsten Mehlig, Paola Russo, Luis A. Moreno, Stefaan De Henauw, Toomas Veidebaum, Dénes Molnár, Michael Tornaritis, Patrizia Risé, Maike Wolters, on behalf of the IDEFICS and I.Family consortia, was originally published Online First without Open Access. After publication in volume 179, issue 10, page 1647–1651 the author decided to opt for Open Choice and to make the article an Open Access publication. Therefore, the copyright of the article has been changed to © The Author(s) 2021 and the article is forthwith distributed under the terms of the Creative Commons Attribution 4.0 International License, which permits use, sharing, adaptation, distribution and reproduction in any medium or format, as long as you give appropriate credit to the original author(s) and the source, provide a link to the Creative Commons licence, and indicate if changes were made. The images or other third party material in this article are included in the article’s Creative Commons licence, unless indicated otherwise in a credit line to the material. If material is not included in the article’s Creative Commons licence and your intended use is not permitted by statutory regulation or exceeds the permitted use, you will need to obtain permission directly from the copyright holder. To view a copy of this licence, visit http://creativecommons.org/licenses/by/4.0.

