# Supplementary material for: Associations of whole blood polyunsaturated fatty acids and insulin resistance among European children and adolescents
Source: Eur J Pediatr. 2020 Apr 8;179(10):1647–51. doi: 10.1007/s00431-020-03636-1 (PMC8463339; doi:10.1007/s00431-020-03636-1)
Supplement: Supplementary file 1 — (DOCX 88 kb) [file 431_2020_3636_MOESM1_ESM.docx]

**Supplementary Material and Tables to the Manuscript:**

**Associations of whole blood polyunsaturated fatty acids and insulin resistance among European children and adolescents**

**Abbreviations**

AA: Arachidonic acid

ALA: Alpha-linolenic acid

BMI: Body Mass Index

DHA: Docosahexaenoic acid

EPA: Eicosapentaenoic acid

HOMA: Homeostasis Model Assessment for Insulin Resistance

IDEFICS study: Identification and prevention of dietary- and lifestyle-induced health effects in children and infants study

IR: Insulin resistance

ISCED: Internationals Standard Classification of Education

LA: Linoleic acid

PUFA: Polyunsaturated fatty acids

MUFA: Monounsaturated fatty acids

SFA: Saturated fatty acids

### *MATERIALS AND METHODS*

### Quality management

### For all study procedures, detailed standard operating procedures were provided to all partners. The central training of field staff and conduction of site visits during the field work were important measures of the quality control in order to make sure that the different sites complied with the standard operating procedures. Additionally, for all blood samples, the processing and laboratory analyses were performed centrally.

### Fatty acid assessment

Blood was obtained by collecting a drop of blood from a fingertip or by venipuncture and immediately applied to a butylated hydroxytoluene (BHT)-prepared test strip. The whole blood fatty acids were separated by gas-liquid chromatography (GC) without prior extraction of total lipids, and directly after derivatization to the fatty acid methyl esters (FAME)^1,2^. The method has been validated by a couple of laboratories^2,3,4^. Fatty acids with cis configuration with a C16-C24 chain length were measured. In the present study, the n-6 PUFA AA and the n-3 PUFA ALA, EPA and DHA as well as the sum of EPA and DHA (EPA+DHA) were considered. The PUFA proportions are expressed as weight percentage of all FA detected (% wt/wt).

### Insulin resistance assessment

At T0 and T1, blood of either venous or capillary origin was used to assess fasting glucose by point-of-care analysis using the Cholestech LDX analyser (Cholestech, Cholestech Corp., Hayward, CA, USA). At T3, serum fasting glucose was measured using an enzymatic UV test (Cobas c701, Roche Diagnostics GmbH, Mannheim, Germany). Insulin was assessed through electrochemiluminescence immunoassay (Roche Modular System, Mannheim, Germany). HOMA was calculated as fasting insulin (μǀU/ml) x fasting glucose (mmol/l)/ 22.5^5^. Age- and sex-specific reference values for blood glucose, insulin and HOMA in children have already been published using IDEFICS data.^6^ Applying the same methods, these references were extended based on the IDEFICS/I.Family cohort data and used in the current analysis to also include older age groups from the I.Family study. As the laboratory method for glucose changed from T0/T1 to T3, separate reference curves were estimated, depending on the assessment method used. HOMA z-scores of T0, T1 and T3 were used as the outcome in the statistical analyses. For the description of the characteristics of the study population, children were divided by their HOMA status: Children with a HOMA greater or equal to the 90th percentile (≥P90) were considered to be insulin resistant or at increased risk for IR.

### Anthropometric data

Body height was measured with a calibrated stadiometer (Seca 225/213 stadiometer, Birmingham, UK) to the nearest 0.1 cm. Weight was assessed on a calibrated Tanita scale (Tanita Europe GmbH, Sindelfingen, Germany) accurate to 0.1 kg in a fasting state, with children wearing light underwear. Weight (kg) divided by height (m) squared was calculated to determine the Body Mass Index (BMI). BMI z-scores were calculated according to Cole & Lobstein.^7^

### Covariates

The following covariates were considered in accordance with previous literature: age (continuous), sex, country of residence (6 categories: Estonia, Germany, Hungary, Italy, Spain, Sweden), maximum ISCED^8^ (International Standard Classification of Education) level of parents (three categories: low level: ISCED 0, 1, 2; medium: ISCED 3, 4; high: ISCED 5, 6.), family history of diabetes mellitus type 2 (yes vs. no; as reported for biological parents and siblings at any of the three time points), BMI z-scores, birth weight and pubertal stage (pubertal, pre-pubertal, no information available; definition based on first menstrual period in girls and on voice change in boys), the consumption frequency of sugar/refined carbohydrates (times per day) assessed using a food frequency questionnaire, being a member of a sports club (yes vs. no, as an indicator for physical activity (PA)), time spent using audio-visual media (hours per week) as a proxy for sedentary behavior time. As there was an embedded community- and setting-oriented intervention program in the IDEFICS study, a binary variable indicating either intervention or control regions was added to all models.

The weight percentages of our focus PUFA also depend on the individual proportions of different lipid classes in plasma, erythrocytes, HDL and LDL, in which saturated (SFA) and monounsaturated fatty acids (MUFA) are the major fatty acids with different distributions.^3^ Therefore, the weight percentages of total SFA and total MUFA of total fatty acids were also included as covariates.

***Statistical analyses***

Mixed effect models were used to assess the association between different PUFA measured at baseline and repeated measurements of HOMA z-scores at baseline, 2- and 6-year follow-up. Such models can handle unbalanced data (children measured at different ages and with different numbers of repeated measurements) under a missing at random assumption.^9^ To investigate how associations change over time, a variable “time since baseline” was constructed, which takes the values 0 for the T0 survey, approximately two years for T1, and six years for T3. A linear as well as a quadratic term was added. The model was built as follows.

$${HOMA}_{i,j} = \left( \beta_{0} + u_{i,0} \right)+ \left( \beta_{1} + u_{i,1} \right)t{ime_{since_{T0}}}_{i,j}+ \beta_{2}*\left( t{ime_{since_{T0}}}_{i,j} \right)^{2}+ \beta_{3}{T0_{exposure}}_{i} +\beta_{4}{T0_{exposure}}_{i}* t{ime_{since_{T0}}}_{i,j}+ \beta_{5}{T0\_exposure}_{i}*\left( t{ime_{since_{T0}}}_{i,j} \right)^{2}+ \beta_{covars}{T0\_covariates}_{i} + \varepsilon_{i,j}$$

- HOMA*_i,j_* describes the individual’s outcome value *i* measured at time *j* (i=1,…,N where N describes the number of participants in the study; *j*=0,1,2 denotes the survey wave),
- *β*_0_ represents the intercept, *u_i_*_,0_ a random participant-specific intercept,
- *β*_1_, *β*_2_ indicate the annual linear and quadratic changes in average HOMA levels since baseline,
- *u_i_*_,1_ is a participant-specific parameter describing the individual’s change of HOMA over time,
- *β*_3_ represents the effect of the exposure at T0 on HOMA at T0,
- *β*_4,_ *β*_5_ denote the linear and quadratic annual changes in the effect of the FA exposure at baseline on HOMA,
- *β_covars_* stands for effect estimates of baseline covariates in the model (e.g. if age and sex were included as covariables, *β_covars_* would refer to the two effect estimates *β_age_* and *β_sex_* for age and sex. *ε_i,j_* is the error term for participant *i* at time *j*.

For the random effects, an unstructured covariance matrix was chosen. The basic model was adjusted for age (continuous), sex, country of residence (six categories: Estonia, Germany, Hungary, Italy, Spain, Sweden) and intervention vs. control region (binary dummy variable, because of an embedded community- and setting-oriented intervention in the IDEFICS study). In line with previous literature, the fully adjusted models were additionally adjusted for maximum ISCED^10^ (International Standard Classification of Education) level of parents (three categories: low level: ISCED 0-2; medium: ISCED 3&4, high: ISCED 5&6), family history of diabetes (yes vs. no), BMI z-score, birth weight and pubertal status (pubertal, pre-pubertal, no information available; definition based on first menstrual period in girls and on voice change in boys), consumption frequency of sugar/refined carbohydrates (times per day), being a member of a sports club (yes vs. no - as an indicator for physical activity), time spent with audio-visual media (hours per week - as a proxy for sedentary behavior time). A p value of 0.01 was considered to be statistically significant.

***DISCUSSION***

### Measurement methods potentially influencing the associations

Differences in measurement methods and in the nature of the biosample for the assessment of PUFA status (whole blood, erythrocytes, plasma) or IR/IS (HOMA, hyperinsulinemic-euglycemic clamp technique) may have contributed towards the conflicting results we observed compared to other studies. As is usually done in large epidemiological studies, we used HOMA as a simple method. The gold standard to measure insulin sensitivity is however the hyperinsulinemic-euglycemic clamp technique. Study results have been observed to vary depending on the insulin resistance or sensitivity index applied.^11,12^ While fatty acids measured in plasma or serum reflect short-term fat intake and are thus subject to stronger variations depending on recent dietary intake, erythrocytes reflect long-term intake, and adipose tissue with half-life period of about 1-2 years very long-term intake. Whole blood as used in our study consists of 46% of circulating cells by volume, mostly erythrocytes,^13^ whose cell membrane phospholipids are rich in long-chain PUFA such as EPA and DHA. Therefore, whole blood and also erythrocytes contain higher long-chain PUFA (DHA, AA) and lower C:18-PUFA (LA, ALA) proportions than serum or plasma.^2^ Whole blood includes fatty acids from all lipid classes and has been shown to be representative for the total fatty acid status.^3^

**References**

1. Wolters M, Pala V, Russo P, et al. Associations of whole blood n-3 and n-6 polyunsaturated fatty acids with blood pressure in children and adolescents - results from the idefics/i.Family cohort. *PloS one.* 2016;11(11):e0165981.

2. Marangoni F, Colombo C, Galli C. A method for the direct evaluation of the fatty acid status in a drop of blood from a fingertip in humans: Applicability to nutritional and epidemiological studies. *Analytical biochemistry.* 2004;326(2):267-272.

3. Rise P, Eligini S, Ghezzi S, Colli S, Galli C. Fatty acid composition of plasma, blood cells and whole blood: Relevance for the assessment of the fatty acid status in humans. *Prostaglandins, leukotrienes, and essential fatty acids.* 2007;76(6):363-369.

4. Rise P, Tragni E, Ghezzi S, et al. Different patterns characterize omega 6 and omega 3 long chain polyunsaturated fatty acid levels in blood from italian infants, children, adults and elderly. *Prostaglandins, leukotrienes, and essential fatty acids.* 2013;89(4):215-220.

5. Matthews DR, Hosker JP, Rudenski AS, Naylor BA, Treacher DF, Turner RC. Homeostasis model assessment: Insulin resistance and beta-cell function from fasting plasma glucose and insulin concentrations in man. *Diabetologia.* 1985;28(7):412-419.

6. Peplies J, Jimenez-Pavon D, Savva SC, et al. Percentiles of fasting serum insulin, glucose, hba1c and homa-ir in pre-pubertal normal weight european children from the idefics cohort. *International journal of obesity (2005).* 2014;38 Suppl 2:S39-47.

7. Cole TJ, Lobstein T. Extended international (iotf) body mass index cut-offs for thinness, overweight and obesity. *Pediatric obesity.* 2012;7(4):284-294.

8. Nations U. International standard classification of education. Published 1997. Accessed.

9. Cnaan A, Laird NM, Slasor P. Using the general linear mixed model to analyse unbalanced repeated measures and longitudinal data. *Statistics in medicine.* 1997;16(20):2349-2380.

10. Nations U. International standard classification of education 2011. *UNESCO Institute for Statistics, Montreal, Canada.* 2011.

11. Malita FM, Messier V, Lavoie JM, Bastard JP, Rabasa-Lhoret R, Karelis AD. Comparison between several insulin sensitivity indices and metabolic risk factors in overweight and obese postmenopausal women: A monet study. *Nutrition, metabolism, and cardiovascular diseases : NMCD.* 2010;20(3):173-179.

12. Dasgupta R, Anoop S, Venkatesan P, Inbakumari M, Finney G, Thomas N. Differential performance of surrogate indices of fasting insulin resistance in low-birthweight and normal-birth weight cohorts: Observations from hyperinsulinaemic-euglycaemic clamp studies in young, asian indian males. *Diabetes & metabolic syndrome.* 2019;13(1):770-775.

13. Hodson L, Skeaff CM, Fielding BA. Fatty acid composition of adipose tissue and blood in humans and its use as a biomarker of dietary intake. *Prog Lipid Res.* 2008;47(5):348-380.

**Supplementary Tables**

**Supplementary Table 1** Characteristics of the study population by percentile category (P) of HOMA (<90^th^ versus ≥90^th^) at T0 (baseline), T1 (after 2 years of follow-up) and T3 (after 6 years of follow-up)

|  | **T0** | | | | **T1** | | | | **T3** | | | |
| --- | --- | --- | --- | --- | --- | --- | --- | --- | --- | --- | --- | --- |
|  | **HOMA <P90** | | **HOMA ≥P90** | | **HOMA <P90** | | **HOMA ≥P90** | | **HOMA <P90** | | **HOMA ≥P90** | |
|  | **N** | **%** | **N** | **%** | **N** | **%** | **N** | **%** | **N** | **%** | **N** | **%** |
| All | 554 | 80.2 | 137 | 19.8 | 408 | 73.1 | 150 | 26.9 | 250 | 74.4 | 86 | 25.6 |
| Male | 281 | 81.2 | 65 | 18.8 | 213 | 76.1 | 67 | 23.9 | 127 | 76.5 | 39 | 23.5 |
| Female | 273 | 79.1 | 72 | 20.9 | 195 | 70.1 | 83 | 29.9 | 123 | 72.4 | 47 | 27.6 |
| 2≤age<6 | 216 | 85.7 | 36 | 14.3 | 154 | 79.0 | 41 | 21.0 | 103 | 73.6 | 37 | 26.4 |
| 6≤age<10 | 338 | 77.0 | 101 | 23.0 | 254 | 70.0 | 109 | 30.0 | 147 | 75.0 | 49 | 25.0 |
| Low education level | 64 | 76.2 | 20 | 23.8 | 45 | 62.5 | 27 | 37.5 | 20 | 62.5 | 12 | 37.5 |
| Medium education level | 274 | 79.7 | 70 | 20.3 | 190 | 69.3 | 84 | 30.7 | 127 | 67.9 | 60 | 32.1 |
| High education level | 216 | 82.1 | 47 | 17.9 | 173 | 81.6 | 39 | 18.4 | 103 | 88.0 | 14 | 12.0 |
| Thin | 45 | 97.8 | 1 | 2.2 | 35 | 94.6 | 2 | 5.4 | 21 | 91.3 | 2 | 8.7 |
| Normal weight | 333 | 91.7 | 30 | 8.3 | 254 | 89.1 | 31 | 10.9 | 150 | 84.3 | 28 | 15.7 |
| Overweight | 111 | 72.5 | 42 | 27.5 | 81 | 64.3 | 45 | 35.7 | 45 | 58.4 | 32 | 41.6 |
| Obese | 65 | 50.4 | 64 | 49.6 | 38 | 34.5 | 72 | 65.5 | 34 | 58.6 | 24 | 41.4 |
| Pre-pubertal | 171 | 80.3 | 42 | 19.7 | 119 | 82.1 | 26 | 17.9 | 118 | 72.0 | 46 | 28.0 |
| Pubertal | 163 | 79.9 | 41 | 20.1 | 100 | 71.4 | 40 | 28.6 | 128 | 78.0 | 36 | 22.0 |
| Missing | 220 | 80.3 | 54 | 19.7 | 189 | 69.2 | 84 | 30.8 | 4 | 50.0 | 4 | 50.0 |
| Member of a sports club | 296 | 80.4 | 72 | 19.6 | 215 | 74.1 | 75 | 25.9 | 145 | 77.1 | 43 | 22.9 |
| No sports club membership | 258 | 79.9 | 65 | 20.1 | 193 | 72.0 | 75 | 28.0 | 105 | 70.9 | 43 | 29.1 |
| No familial diabetes | 530 | 80.4 | 129 | 19.6 | 393 | 73.6 | 141 | 26.4 | 238 | 74.6 | 81 | 25.4 |
| Familial history of diabetes | 24 | 75.0 | 8 | 25.0 | 15 | 62.5 | 9 | 37.5 | 12 | 70.6 | 5 | 29.4 |
|  | **HOMA <P90** | | **HOMA ≥P90** | | **HOMA <P90** | | **HOMA ≥P90** | | **HOMA <P90** | | **HOMA ≥P90** | |
|  | **Mean** | **SD** | **Mean** | **SD** | **Mean** | **SD** | **Mean** | **SD** | **Mean** | **SD** | **Mean** | **SD** |
| HOMA | 0.81 | 0.43 | 2.52 | 1.76 | 0.90 | 0.63 | 1.80 | 1.79 | 1.09 | 1.41 | 1.37 | 1.08 |
| HOMA z-score | -0.07 | 0.89 | 1.81 | 0.49 | 0.01 | 1.05 | 1.03 | 0.99 | 0.18 | 1.13 | 0.71 | 1.05 |
| Alpha-linolenic acid, ALA | 0.20 | 0.08 | 0.21 | 0.10 | 0.20 | 0.09 | 0.20 | 0.08 | 0.22 | 0.09 | 0.20 | 0.08 |
| Arachidonic acid, AA^§^ | 7.43 | 1.29 | 7.43 | 1.53 | 7.41 | 1.31 | 7.68 | 1.42 | 7.34 | 1.23 | 7.61 | 1.34 |
| Eicosapentaenoic acid, EPA^§^ | 0.28 | 0.12 | 0.27 | 0.13 | 0.28 | 0.12 | 0.27 | 0.11 | 0.29 | 0.12 | 0.26 | 0.10 |
| Docosahexaenoic acid, DHA^§^ | 1.20 | 0.42 | 1.16 | 0.49 | 1.23 | 0.44 | 1.24 | 0.45 | 1.18 | 0.42 | 1.16 | 0.43 |
| Sum of EPA+DHA^§^ | 1.48 | 0.48 | 1.44 | 0.56 | 1.50 | 0.50 | 1.51 | 0.49 | 1.47 | 0.48 | 1.41 | 0.48 |
| Total SFA^§^ | 44.49 | 1.93 | 44.30 | 2.06 | 44.42 | 1.83 | 44.22 | 2.18 | 44.49 | 2.04 | 44.64 | 2.07 |
| Total MUFA^§^ | 25.00 | 2.28 | 24.78 | 2.56 | 24.95 | 2.22 | 24.69 | 2.44 | 25.21 | 2.27 | 25.07 | 2.24 |
| Sugar/refined carbohydrates, consumption frequency [times per day] | 4.04 | 2.62 | 3.68 | 2.21 | 4.00 | 2.65 | 3.66 | 2.17 | 4.19 | 2.82 | 3.69 | 2.11 |
| Time spent with audio-visual media [hours per week] | 11.94 | 7.93 | 13.92 | 8.29 | 12.06 | 7.74 | 13.70 | 8.82 | 12.48 | 7.69 | 11.73 | 6.87 |
| Birth weight [g] | 3358 | 563 | 3433 | 478 | 3381 | 555 | 3348 | 537 | 3364 | 547 | 3346 | 594 |
| BMI z-score^‡^ | 0.64 | 1.25 | 1.97 | 1.18 | 0.60 | 1.21 | 1.90 | 1.14 | 0.58 | 1.32 | 1.49 | 1.24 |
| BMI [kg/m^2^] | 17.06 | 2.75 | 20.65 | 3.79 | 16.94 | 2.70 | 20.46 | 3.58 | 17.05 | 2.95 | 18.82 | 3.21 |

^‡^ Cole & Lobstein 2012^41^

^§^weight percentage of all FA detected (% wt/wt)

Abbreviations: BMI, body mass index; HOMA, Homeostasis Model Assessment for Insulin Resistance; MUFA, monounsaturated fatty acids; SFA, saturated fatty acids

Supplementary Table 2 Associations of polyunsaturated fatty acids measured at baseline with HOMA z-scores at baseline, after two years and after six years of follow-up estimated on basic and fully adjusted mixed-effect models stratified by sex

| **Fatty acid** | **Time since baseline in years** | **Basic** | | | |  | **Full Adjustment**^†^ | | |
| --- | --- | --- | --- | --- | --- | --- | --- | --- | --- |
|  |  | **Male** | | **Female** | | **Male** | | **Female** | |
|  |  | **β** | ***p* value** | **β** | ***p* value** | **β** | ***p* value** | **β** | ***p* value** |
| 20:4n-6, AA | 0 | -0.032 | 0.5549 | -0.061 | 0.2446 | -0.135 | 0.0189 | -0.040 | 0.4628 |
|  | 2 | 0.081 | 0.1242 | 0.066 | 0.2413 | -0.044 | 0.4248 | 0.053 | 0.1161 |
|  | 6 | -0.011 | 0.9135 | 0.064 | 0.3378 | 0.106 | 0.3202 | 0.070 | 0.2729 |
| 18:3n-3, ALA | 0 | 1.174 | 0.1391 | **1.977** | **0.0058** | 0.834 | 0.2611 | 0.940 | 0.1149 |
|  | 2 | 0.706 | 0.4568 | -0.138 | 0.8650 | 0.878 | 0.3498 | -1.008 | 0.0971 |
|  | 6 | 0.656 | 0.6189 | -0.777 | 0.3896 | 0.270 | 0.8223 | -1.054 | 0.2056 |
| 20:5n-3, EPA | 0 | 1.203 | 0.0372 | **1.238** | **0.0057** | 0.553 | 0.3497 | 0.793 | 0.0440 |
|  | 2 | 0.728 | 0.2807 | 0.503 | 0.3421 | 0.319 | 0.5734 | 0.339 | 0.4606 |
|  | 6 | -1.348 | 0.2352 | -0.327 | 0.6974 | -2.028 | 0.0593 | -0.550 | 0.4940 |
| 22:6n-3, DHA | 0 | 0.182 | 0.2493 | -0.160 | 0.3066 | 0.097 | 0.5337 | -0.003 | 0.9796 |
|  | 2 | 0.246 | 0.1204 | 0.014 | 0.9303 | 0.159 | 0.2898 | 0.174 | 0.2092 |
|  | 6 | -0.318 | 0.2461 | 0.347 | 0.1169 | -0.467 | 0.0873 | 0.422 | 0.0497 |
| 20:5n-3+22:6n-3 (sum of EPA+DHA) | 0 | 0.198 | 0.1532 | -0.051 | 0.7016 | 0.104 | 0.4504 | 0.048 | 0.6749 |
|  | 2 | 0.225 | 0.1067 | 0.038 | 0.7840 | 0.141 | 0.2853 | 0.156 | 0.1849 |
|  | 6 | -0.311 | 0.2015 | 0.250 | 0.2089 | -0.454 | 0.0617 | 0.299 | 0.1244 |

^†^ The basic model was adjusted for age, sex, country of residence and control vs. intervention region. The fully adjusted model was furthermore adjusted for birth weight, BMI z-score, pubertal status, family history of diabetes type 2, being a member of a sports club, consumption frequency of sugar/refined carbohydrates, time spent with audio-visual media, , maximum ISCED level of parents, weight percentage of the sum of total SFA and total MUFA of total fatty acids.

As highlighted in bold, a *p* value of 0.01 was used as the level of statistical significance.

Abbreviations: AA, arachidonic acid; ALA, α-linolenic acid; DHA, docosahexaenoic acid; EPA, eicosapentaenoic acid; HOMA, Homeostasis Model Assessment for Insulin Resistance

Supplementary Table 3 Associations of polyunsaturated fatty acids measured at baseline with HOMA z-scores at baseline, after two years and after six years of follow-up estimated on basic and fully adjusted mixed-effect models stratified by weight status.

| **Fatty acid** | **Time since baseline in years** | **Basic** | | | | **Full Adjustment**^†^ | | | |
| --- | --- | --- | --- | --- | --- | --- | --- | --- | --- |
|  |  | **Thin/normal** | | **Overweight** | | **Thin/normal** | | **Overweight** | |
|  |  | **β** | ***p* value** | **β** | ***p* value** | **β** | ***p* value** | **β** | ***p* value** |
| 20:4n-6, AA | 0 | **-0.131** | **0.0063** | 0.001 | 0.9835 | -0.134 | 0.0169 | -0.039 | 0.5215 |
|  | 2 | 0.027 | 0.5852 | 0.042 | 0.3583 | 0.025 | 0.6451 | 0.004 | 0.9427 |
|  | 6 | -0.027 | 0.6357 | 0.128 | 0.2905 | -0.029 | 0.6546 | 0.071 | 0.5700 |
| 18:3n-3, ALA | 0 | 0.933 | 0.2232 | 0.790 | 0.1787 | 1.199 | 0.1185 | 0.784 | 0.1972 |
|  | 2 | -0.223 | 0.8019 | -0.435 | 0.5589 | 0.037 | 0.9666 | -0.417 | 0.5644 |
|  | 6 | 0.093 | 0.9208 | -1.362 | 0.2570 | 0.047 | 0.9603 | -1.355 | 0.2641 |
| 20:5n-3, EPA | 0 | 0.822 | 0.1065 | 0.231 | 0.6014 | 1.183 | 0.0192 | 0.180 | 0.6925 |
|  | 2 | 0.389 | 0.4586 | -0.107 | 0.8328 | 0.672 | 0.1934 | -0.148 | 0.7790 |
|  | 6 | -0.614 | 0.4254 | -0.726 | 0.1839 | -0.403 | 0.6048 | -2.080 | 0.0736 |
| 22:6n-3, DHA | 0 | -0.078 | 0.5821 | 0.037 | 0.7750 | -0.014 | 0.9300 | -0.006 | 0.9684 |
|  | 2 | 0.104 | 0.4522 | 0.191 | 0.1804 | 0.173 | 0.2351 | 0.149 | 0.3478 |
|  | 6 | -0.044 | 0.8014 | -0.049 | 0.8795 | -0.013 | 0.9470 | -0.105 | 0.7507 |
| 20:5n-3+22:6n-3 (sum of EPA+DHA) | 0 | -0.014 | 0.9081 | 0.042 | 0.7091 | 0.063 | 0.6331 | 0.005 | 0.9676 |
|  | 2 | 0.102 | 0.4059 | 0.145 | 0.2342 | 0.179 | 0.1583 | 0.110 | 0.4182 |
|  | 6 | -0.067 | 0.6779 | -0.165 | 0.5644 | -0.022 | 0.9001 | -0.205 | 0.4789 |

^†^ The basic model was adjusted for age, sex, country of residence and control vs. intervention region. The fully adjusted model was furthermore adjusted for birth weight, BMI z-score, pubertal status, family history of diabetes type 2, being a member of a sports club, consumption frequency of sugar/refined carbohydrates, time spent with audio-visual media, , maximum ISCED level of parents, weight percentage of the sum of total SFA and total MUFA of total fatty acids.

As highlighted in bold, a *p* value of 0.01 was used as the level of statistical significance.

Abbreviations: AA, arachidonic acid; ALA, α-linolenic acid; DHA, docosahexaenoic acid; EPA, eicosapentaenoic acid; HOMA, Homeostasis Model Assessment for Insulin Resistance
